# Identification of factors related to behaviors associated with musculoskeletal pain among elementary students

**DOI:** 10.1186/s12891-021-04413-3

**Published:** 2021-06-08

**Authors:** Forouzan Rezapur-Shahkolai, Elham Gheysvandi, Akram Karimi-Shahanjarini, Leili Tapak, Rashid Heidarimoghadam, Iman Dianat

**Affiliations:** 1 Department of Public Health, School of Public Health, Hamadan University of Medical Sciences, Hamadan, Iran; 2 Social Determinants of Health Research Center, Hamadan University of Medical Sciences, Hamadan, Iran; 3 Research Center for Health Sciences, Hamadan University of Medical Sciences, Hamadan, Iran; 4 Department of Biostatistics, School of Public Health, Hamadan University of Medical Sciences, Hamadan, Iran; 5 Modeling of Non-communicable diseases research center, Hamadan University of Medical Sciences, Hamadan, Iran; 6 Department of Ergonomics, School of Public Health, Hamadan University of Medical Sciences, Hamadan, Iran; 7 Department of Occupational Health and Ergonomics, Faculty of Health, Tabriz University of Medical Sciences, Tabriz, Iran

**Keywords:** Sitting posture, Backpack carrying, School health, Health promotion model

## Abstract

**Background:**

Musculoskeletal pains are among evident health problems in children and adolescents. Backpack carrying behaviors and the sitting postures are among behavioral factors associated with musculoskeletal pain in schoolchildren. Therefore, this study aims to identify the factors related to these important musculoskeletal behaviors, using Health Promotion Models.

**Methods:**

In this cross-sectional study, a questionnaire was created based on PRECEDE Model and Health Belief Model and was administered to 673 Iranian students, whom were selected randomly from elementary schools of Hamadan, Iran, in 2018.

**Results:**

The findings of the study revealed that proper sitting postures and backpack carrying were 42 and 33%, respectively. The findings also showed that predisposing factors including perceived susceptibility (*p* < 0.05, β = 0.219), perceived severity (*p* < 0.05, β = 0.166), perceived barriers (*p* < 0.05, β = − 0.191), perceived self-efficacy (*p* < 0.05, β = 0.188) and also enabling factors (*p* < 0.05, β = 0.329) were significantly related to sitting behaviors. Moreover, backpack carrying behaviors had significant relationships with predisposing factors of perceived susceptibility (*p* < 0.05, β = 0.198), perceived barriers (*p* < 0.05, β = − 0.258), perceived self-efficacy (*p* < 0.05, β = 0.185) and reinforcing factors (*p* < 0.05, β = 0.208).

**Conclusions:**

It seems necessary for future preventive programs to take factors of musculoskeletal pains among children and adolescents into account.

## Background

Musculoskeletal (MSK) pains are among prevalent pains affecting muscles, bones, joints, ligaments, and tendon [1] of which back pain, neck pain and other musculoskeletal pains rank 1st, 4th, and 10th respectively among health problems in years lived with disability [2]. Childhood and adolescence are of the highest significance in developing musculoskeletal system. Physical problems in these periods may be a predicting factor for irreversible disorders in adulthood because bones and muscles develop in earlier life stages [3]. MSK pains have been reported in 40% of youths [4], influencing their function in daily activities like studying, exercising, and social participation which in turn lead to health burdens and life costs [5]. As a result, identifying dimensions and risk factors of initial musculoskeletal pains provides great opportunities to formulate effective treatments and efforts to prevent the pains [6].

Musculoskeletal pain is a multifactorial phenomenon being influenced by lifestyle factors, work and age. With regard to MSK pain in children, some outstanding factors are school furniture improper to students’ ergonomics, bad postures of sitting or carrying heavy school bags (more than 10% of body weight) [7–10]. Bad postures refer to deviations from neutral spinal curvature [11]. Research has shown that sitting with twisted trunk, kyphotic sitting, or sitting with flexed neck can add distress and strains to spine and ligaments. Schools environments seem to expose children to many potential risk factors of long bad sittings [12, 13]. Students spend considerable time (about 6 h/D) in schools which require log-time sittings [14]. In addition to bad sitting postures, heavy school bags and carrying those for a long time can have adverse effects on students’ musculoskeletal systems. Carrying wrong heavy backpacks brings fatigue and back pains as well as abnormal spinal curves, scoliosis and malformation of spines [15].

**Fig. 1:**
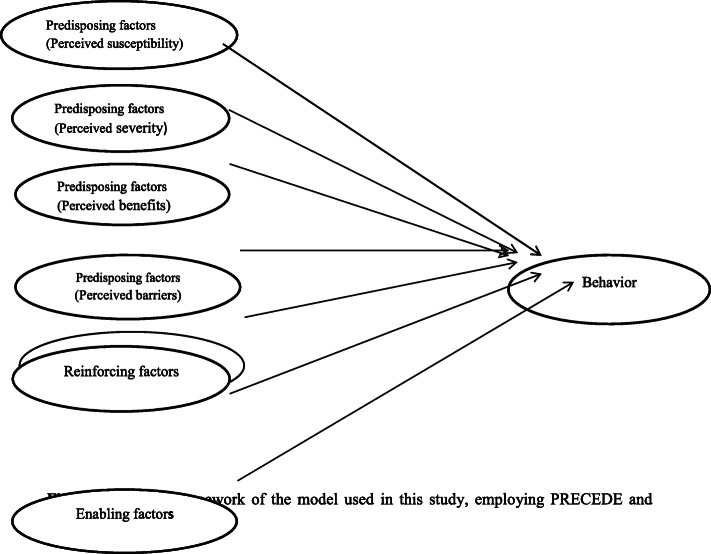
Conceptual framework of the model used in this study, employing PRECEDE and Health Belief Models

The employment of Models and Theories of Health Education and Health Promotion in many different studies has been proven helpful in identifying risk factors, improving behaviors, and preventing health problems [16–21]. However, the research team of the present study found no considerable knowledge on the behaviors related to musculoskeletal pains, especially among children and adolescents. One important model in health promotion programs is PRECEDE model. The present study focused on behavioral educational phase of PRECEDE model. This phase comprises predisposing factors (knowledge, attitudes, perceptions, beliefs), reinforcing factors (family, peer, teacher influences) as well as enabling factors (availability of resources, skills) [22]. Some studies, including the studies, done by the research team of current study, reported high prevalence of musculoskeletal pains and effects of some risk factors and predictors on them [23, 24]. Some of these predictors are knowledge and beliefs of care for back and spine, posture and ergonomics and other cognitive factors like perceived self-efficacy, perceived benefits, perceived barriers and intention behavior [7, 25–28]. Thus, in this study, the constructs of Health Belief Model including perceived susceptibility, perceived severity, perceived benefits, perceived barriers and perceived self-efficacy also were employed (22). Conceptual framework of the PRECEDE model is given in Fig. 1.

This study is part of a larger research project conducted on musculoskeletal pain and its risk factors [23, 24]. We found that backpack carrying behaviors and the sitting postures were among the factors associated with musculoskeletal pain, so in the present study we examined the factors associated with these two important behaviors, using Health Promotion Models including PRECEDE and Health Belief.

## Methods

This cross-sectional study was conducted in Hamadan, Iran, from April to May 2018. The sample consisted of 673 elementary school students selected by multistage random sampling. The sample size was estimated by using the $n=\frac{{\left({z}_{\alpha /2}\right)}^2p\left(1-p\right)}{E^2}$ formula, taking into account the 95% confidence level, 0.95 (1-α = 0.95), the same prevalence that Dianat et al. found in their study [29], with a *p* = 0.28 and an estimation error (E) of maximum 15%, applying a cluster sampling factor of 1.5 and attrition 15%. Therefore, the sample size was estimated as 780 students.

First, a list of elementary schools of Hamadan city was provided. Then, 13 schools were selected based on regions with different socioeconomic status (high, moderate and low). Students were selected by simple random sampling from all grades (1st-6th); one class from each grade. The written informed consent was obtained from all students’ parents before inclusion. There were 780 eligible individuals, of which 673 participated in the study (participation rate = 86.28%).

Data was collected through interviews with students, if they gave informed consent, using a researcher-made questionnaire. The questionnaire was formulated based on the related literature [7, 28, 30, 31] and consisted of two sections: First section included demographic information and second section was designed according to the constructs of PRECEDE Model and Health Belief Model. PRECEDE-based questionnaire consisted of predisposing, reinforcing, and enabling factors, as well as the students’ behaviors. These measures were performed on both sitting and backpack carrying behaviors.

Questions of predisposing factors included constructs of Health Belief Model. The number of the questions on these constructs was 20 for backpack carrying. The model measures perceived susceptibility, severity, benefits, barriers and self-efficacy. Perceived susceptibility refers to the subjective belief that a person may acquire a disease or enter a harmful state as a result of a particular behavior. The perceived severity means the belief in the extent of harm that can result from the acquired disease or a harmful state as a result of a particular behavior. The perceived benefits are defined as the belief in the advantages of the methods suggested for reducing the risk or seriousness of the disease or a harmful state resulting from a particular behavior. The perceived barriers are about the belief concerning actual and imagined costs of performing the suggested behavior. The perceived self-efficacy means confidence in one’s ability to acquire the new behavior.

The reinforcing factors which lead to continuing the proper behaviors by a reward or encouragement were assessed with 3 items.

The enabling factors, including environmental factors like services and resources as well as skills that facilitate the behavior directly or indirectly, were evaluated with 5 items [22]. Finally, the backpack carrying behaviors included 4 items.

The questionnaire for sitting postures consisted of predisposing factors (perceived susceptibility 4 items, perceived severity 4 items, perceived benefits 4 items, perceived barriers 9 items, and perceived self-efficacy 3 items), reinforcing factors with 6 items, enabling factors with 5 items and behavior with 4 items.

All questions were rated at 3-point Likert scale: I disagree (1 score), I partly agree, (2 scores), I agree (3 scores). Scoring was reversal for perceived barrier construct. Backpack carrying and sitting behaviors were measured by 3-point Likert scale (“always”, “sometimes”, and “never”, scored “3”, “2”, and 1, respectively).

The ranges of scores and questions are given in Table 1, for sitting postures and Table 2, for backpack carrying behaviors.

**Table 1:** Validity of the questionnaire and indices of measuring model for sitting behaviors

Constructs and questions	Scoring	Mean ± SD	CVR	S-CVI	Factor loading	Cronbach’s alpha	Composite reliability	AVE	T-value
Predisposing factors (Perceived susceptibility)	4 to 12	7.28 ± 2.13	0.92	≥0.70		0.52	0.76	0.51	
If the sitting posture is not good I’ll have pain in my back, neck and shoulders					0.75				10.57
Long-time sitting (more than 20 min) may cause pain in my back, neck and shoulders					0.78				12.76
I won’t get pain in my back, neck and shoulders because I’m too young.					0.60				7.16
Predisposing factors (Perceived severity)	4 to 12	8.97 ± 2.43	0.95	≥0.70		0.72	0.82	0.55	
Pain in my back, neck and shoulders makes me not attend school.					0.81				13.25
I am not being able to focus in class because of pain in back, neck and shoulders					0.74				13.38
Pain in my back, neck and shoulders is a serious disease.					0.76				11.47
Pain in my back, neck and shoulders makes me take drugs.					0.62				7.61
Predisposing factors (Perceived benefits)	4 to 12	9.55 ± 2.05	0.85	≥0.70		0.60	0. 77	0.54	
If my sitting posture is accurate, I’ll get much less pain in my neck, shoulder and back.					0.83				2.90
If I sit in a proper posture, I’ll have much more concentration while learning something					0.67				2.73
If I sit in a proper posture my family won’t have to pay costs of back, shoulder and neck treatments					0.68				2.15
Predisposing factors (Perceived barriers)	9 to 27	19.18 ± 5.58	0.81	≥0.70		0.90	0.91	0.56	
If I sit well in class, my classmates would make fun of me.					0.73				11.73
If I sit in a proper posture,, I will get tired soon					0.71				9.69
If I sit in a proper posture,, I will get distracted					0.80				16.64
If I sit well in class, I can’t catch up with my friends in doing assignments.					0.82				20.36
Improper furniture wouldn’t let me sit properly.					0.73				14.42
The school has stairs so I can’t use a wheeled backpack.					0.76				16.76
Stretching exercises make mess out of the class.					0.76				17.68
The teacher doesn’t let us doing stretching.					0.74				15.63
I will not get bored at home while doing stretching					0.62				8.23
Predisposing factors (Perceived Self-efficacy)	3 to 9	5.88 ± 2.09	0.86	≥0.70		0.87	0.92	0.79	
I always sit on chairs (furniture) in a good posture.					0.86				26.88
I sit well even if I’m tired.					0.90				37.8
I can sit well for a long time (more than 20 min).					0.90				37.90
Reinforcing factors	6 to 18	10.61 ± 2.73	0.93	≥0.70		0.66	0.81	0.60	
My teachers or school nurses encourage me to sit properly in the class					0.88				16.22
My friends encourage me to sit properly in the class					0.53				2.69
My parents and siblings encourage me to sit properly.					0.87				11.29
Enabling factor**s**	5 to 15	9.21 ± 2.75	0.92	≥0.70		0.68	0.80	0.51	
School furniture is good for proper sitting					0.73				13.97
My teachers and school nurses taught me how to sit well.					0.70				8.87
My friends taught me how to sit well.					0.81				27.78
My parents and siblings taught me how to sit well.					0.58				6.02
Behaviors	4 to 12	7.38 ± 2.07				0.69	0.81	0.51	
I put my forearms on the chair, when I’m sitting.					0.71				9.62
When I’m sitting on the chair, I put my legs on the floor.					0.69				10.89
While sitting, I lean my backrest on the chair and never bend over my book or notebook					0.76				16.26
I do stretching when I get tired of sitting for a long time (after 20 min).					0.70				11.39

**Table 2:** Validity of the questionnaire and indices of measuring model for backpack carrying behaviors

Constructs and questions	Scoring	Mean ± SD	CVR	S-CVI	Factor loading	Cronbach’s alpha	Composite reliability	AVE	T-value
Predisposing factors (Perceived susceptibility)	4 to 12	7.71 ± 2.35	0.95	≥0.70		0.60	0.79	0.56	
If I carry a heavy bag, I’ll get pain in my back, neck and shoulders.					0.81				11.42
If is lift my bag with one hand, my spines would be tilted and my shoulders dropped.					0.75				12.82
I won’t get pain in my back, neck and shoulders because I’m too young.					0.66				8.18
Predisposing factors (Perceived severity)	4 to 12	8.97 ± 2.43	0.95	≥0.70		0.72	0.82	0.54	
Pain in my back, neck and shoulders makes me not attend school.					0.82				18.62
Pain in my neck, back and shoulders makes me not concentrate in class.					0.75				11.95
Pain in my back, neck and shoulders is a serious disease.					0.75				8.69
Pain in my back, neck and shoulders makes me take drugs.					0.61				5.71
Predisposing factors (Perceived benefits)	5 to 15	11.35 ± 2.86	0.84	≥0.70		0.73	0.82	0.54	
If I put on both the shoulder straps of my backpack, it prevents me from back, shoulder and neck pains					0.68				7.17
I won’t get back, shoulder and neck pains if I have a lighter backpack.					0.69				7.56
Carrying a light backpack makes me not hunch out.					0.79				15.25
Tying hip straps of my backpack would bring me healthier neck, back and shoulders.					0.77				14.17
Predisposing factors (Perceived barriers)	3 to 9	6.54 ± 2.25	0.90	≥0.70		0.85	0.91	0.77	
I’m too lazy to put my books in my backpack according to my daily schedule.					0.78				12.38
Tying hip straps of my backpack would make me uncomfortable.					0.93				50.63
My teacher wants me to bring all my books and notebooks every day.					0.92				54.39
Predisposing factors (Perceived self-efficacy)	4 to 12	7.89 ± 2.86	0.85	≥0.70		0.88	0.92	0.74	
I can always tie hip straps of my backpack even if it makes me uncomfortable.					0.86				23.63
I can always put on both two shoulder strap of my backpack to carry it.					0.88				34.42
I can only have books of my schedule; I usually keep extra items out of my bag.					0.86				25.23
I can always put heavier books in the back of the backpack which is nearer to my back.					0.83				19.79
Reinforcing factors	3 to 9	5.02 ± 1.75	0.86	≥0.70		0.58	0.77	0.54	
My teachers or schools nurses encourage me to lift and carry my backpack properly.					0.87				23.73
My friends encourage me to lift and carry my backpack properly.					0.69				7.40
My parents and other siblings encourage me to lift and carry my backpack properly.					0.62				5.69
Enabling factor**s**	5 to 15	8.54 ± 2.31	0.92	≥0.70		0.69	0.81	0.52	
My teachers or schools nurses taught me to lift and carry my backpack properly.					0.71				2.72
My friends taught me to lift and carry my backpack properly.					0.70				2.75
My parents and other siblings taught me to lift and carry my backpack properly.					0.74				2.29
TV programs (Media) taught me to lift and carry my backpack properly					0.73				2.43
Behavior**s**	4 to 12	6.71 ± 2.07				0.66	0.79	0.50	
I always put both two shoulder strap of my backpack.					0.76				13.23
I always tie hip straps of my backpack.					0.47				4.22
To lift my bag, I first put it on my seat first and then keep it on my shoulders					0.79				17.57
I always put heavy books in the back of my backpack.					0.74				13.98

Ten experts of Health education, Health promotion and Ergonomics investigated content validity of the questionnaire. To assess the content validity, the content validity ratio (CVR) and the content validity index (CVI) were used. Scores of 0.7 for CVI and 0.6 and above for CVR [32, 33] were acceptable, shown in Tables 1 and 2. To assess the face validity of questions, 10 elementary students were asked to give their comments of simplicity, clearance, and legibility of them. Ambiguous unclear questions were modified.

In order to assess the reliability of the questionnaire, the internal consistency and a test-retest reliability approach were used. A pilot study with 30 participants of elementary students was conducted to assess the internal consistency. Cronbach’s alpha values were estimated as 0.70 to 0.82 for carrying backpack and 0.70 to 0.87 for sitting posture. In addition, to assess the reliability with test-retest approach, the questionnaire was filled by 30 students and then refilled after a period of 2 weeks. An interclass correlation coefficient (ICC) value of 0.80 or higher shows high reliability, an ICC value between 0.60 and 0.79 shows moderate reliability, and an ICC value less than 0.60 shows poor reliability [34]. In the present study, ICC was 0.71–0.90 for carrying backpack and 0.71–0.89 for sitting posture.

### Data analysis

SPSS version 23 and Structural Equation Modeling (SEM) with PLS version 2 were employed to analyze the data. SEM is an approach that consists of two stages: a measuring model and a structural model [35].

To assess the fit of the measuring models, three criteria of reliability, convergent validity, and divergent validity were used. In the first step, factor loadings of the questions and T-values (Bootstrapping done with 5000 subsample) were examined to assess homogeneity of the questions. Factor loading ≥0.4 values and T-values> 1.96 were considered as significant [36, 37]. In the present study, one question of each of the constructs of behaviors related to backpack carrying was removed for their low factor loading value: perceived susceptibility, perceived benefits and enabling factors. In addition, regarding sitting behaviors, one question of perceived susceptibility construct, one question of perceived benefit construct, one question of enabling factors construct and three questions of reinforcing factors construct due to low factor loadings were deleted. In the next step, to examine the reliability of the instrument, composite reliability was assessed using the Cronbach’s alpha with acceptable threshold of > 0.5 [38] and the Average variance extracted (AVE criterion) was used to assess convergent validity, using a threshold of > 0.5 [36]. After confirming the homogeneity and reliability of the instrument, divergent validity was investigated. Divergent validity was examined by Fornell-Larcker test [37]. Once suitable measurement indicators were confirmed, the analysis proceeded to the structural model step.

This study was approved by the Ethics Committee of Hamadan University of Medical Sciences (approval code: IR.UMSHA.REC.1396.641) and all methods were performed in accordance with the relevant guidelines and regulations.

## Results

The average age of the students participated in the study were 9.68 ± 1.58 for girls and 9.76 ± 1.65 for boys. The average of their weight, height and body mass index was as follow: 34.56 ± 10.90 kg, 138.47 ± 11.94 cm, 17.62 ± 3.38 kg/m2 for girls and 34.54 ± 12.05 kg, 137.76 ± 12.10 cm, 17.72 ± 3.77 kg/m2 for boys (Table 3).

**Table 3:** Sociodemographic and anthropometric measurements of participants

Variables	N (%)	Mean ± SD
Age (year)		
Boys	–	9.76 ± 1.65
Girls	–	9.68 ± 1.58
Gender		
Boys	305 (45.3)	–
Girls	368 (54.7)	–
Socioeconomic status		
High	210 (31.2)	–
Moderate	248 (36.8)	–
low	215 (31.9)	–
Weight (kg)		
Boys	–	34.54 ± 12.05
Girls	–	34.56 ± 10.90
Height (cm)		
Boys	–	137.76 ± 12.10
Girls	–	138.47 ± 11.94
BMI (kg/m^2^)		
Boys	–	17.72 ± 3.77
Girls	–	17.62 ± 3.38

The rate of proper sitting postures in students was 42%. Independent variables predicted 70% of sitting behaviors variances (R^2^ = 0.70). Here, the enabling factors seemed to be the strongest predictors. The rate of proper backpack carrying behaviors was 33%. Independent variables determined 53% of variances related to backpack carrying behaviors (R^2^ = 0.53). Here, the perceived barriers were the strongest predictors.

### Measurement model

The loading factors for the items on each construct were higher than loadings with all the remaining constructs (the cross-loadings), and the AVE squared root of any construct was higher than its correlation values with other constructs (Fornell and Larcker test) [39]. These results support discriminant validity at the latent variables level. (Table 4 for sitting postures, and Table 5 for backpack carrying behavior).

**Table 4:** Discriminate validity of Constructs-Fornell-Larcker criterion for behaviors of sitting posture using PROCEED and Health Belief Models

Constructs	1	2	3	4	5	6	7	8
Predisposing factors (Perceived barriers)	0.748							
Behaviors	−0.624	0.720						
Predisposing factors (Perceived benefits)	−0.106	0.151	0.734					
Enabling factors	−0.479	0.706	0.108	0.714				
Reinforcing factors	−0.146	0.270	0.094	0.353	0.774			
Predisposing factors (Perceived Self-efficacy)	−0.640	0.661	0.146	0.536	0.252	0.891		
Predisposing factors (Perceived severity)	−0.332	0.517	0.105	0.430	0.261	0.366	0.741	
Predisposing factors (Perceived susceptibility)	−0.417	0.592	−0.090	0.482	0.185	0.470	0.309	0.720

**Table 5:** Discriminate validity of Constructs-Fornell-Larcker criterion for behaviors of backpack carrying using PROCEED and Health Belief Models

Constructs	1	2	3	4	5	6	7	8
Predisposing factors (Perceived barriers)	0.881							
Behaviors	−0.620	0.707						
Predisposing factors (Perceived benefits)	−0.420	0.402	0.738					
Enabling factors	−0.193	0.120	0.033	0.721				
Reinforcing factors	−0.541	0.562	0.332	0.251	0.738			
Predisposing factors (Perceived Self-efficacy)	−0.584	0.576	0.507	0.166	0.524	0.864		
Predisposing factors (Perceived severity)	−0.418	0.380	0.415	0.142	0.406	0.426	0.740	
Predisposing factors (Perceived susceptibility)	−0.533	0.547	0.311	0.169	0.485	0.475	0.315	0.748

### Structural model

**Fig. 2:**
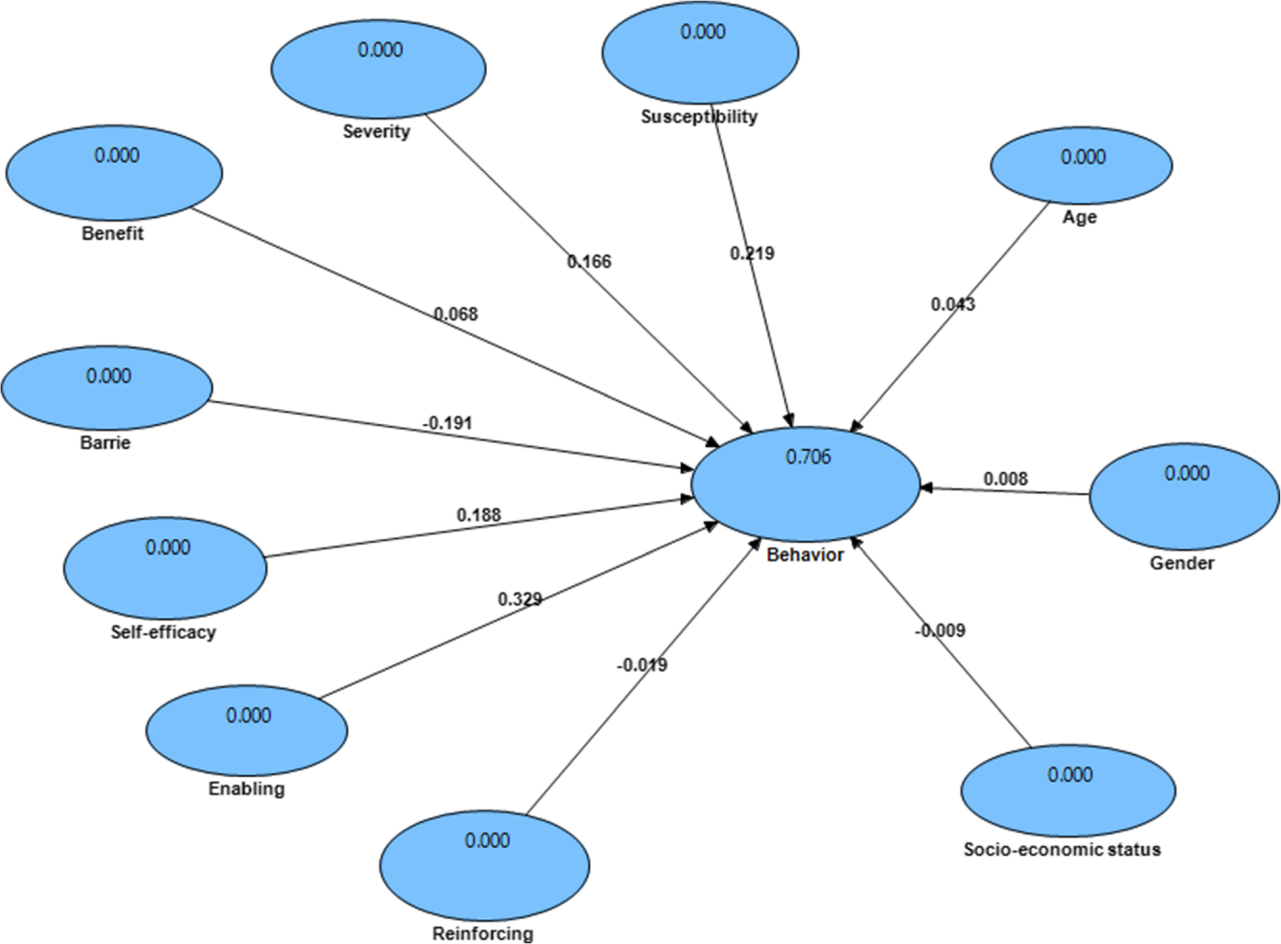
Structural model of sitting behaviors in the prediction of path coefficients

As indicated in Table 6, based on path analysis results, among predisposing factors for sitting behaviors, the perceived susceptibility (*p* < 0.05, β = 0.219), perceived severity (*p* < 0.05, β = 0.166), perceived barriers (*p* < 0.05, β = − 0.191), perceived self-efficacy (*p* < 0.05, β = 0.188) and enabling factors (*p* < 0.05, β = 0.329) were significantly related to sitting behaviors. However, perceived benefits (*p* > 0.05, β = 0.068) of predisposing factors as well as reinforcing factors (*p* > 0.05, β = − 0.019), age (*p* > 0.05, β = 0.043), gender (*p* > 0.05, β = 0.008) and socio-economic status (*p* > 0.05, β = − 0.009) had no significant relationships with sitting behaviors (Fig. 2).

**Table 6:** Indices of structural model of behaviors related to sitting postures and backpack carrying

Sitting Posture				Backpack carrying		
Relationship	Path coefficient	t-value	Effect size (f^**2**^)^**a**^	Path coefficient	t-value	Effect size (f^**2**^)^a^
Perceived susceptibility → Behaviors	0.219	3.08	0.105	0.198	2.26	0.053
Perceived severity → Behaviors	0.166	2.37	0.06	0.025	0.345	0.002
Perceived benefits → Behaviors	0.068	1.02	0.01	0.049	0.602	0.002
Perceived barriers → Behaviors	−0.191	2.48	0.06	−0.258	2.46	0.072
Perceived self-efficacy → Behaviors	0.188	2.40	0.05	0.185	2.11	0.036
Reinforcing factors → Behaviors	−0.019	0.289	0	0.208	2.22	0.055
Enabling factor**s** → Behaviors	0.329	4.71	0.2	−0.056	0.652	0.006
Age → Behaviors	0.043	0.795	0.006	0.068	1.03	0.008
Gender → Behaviors	0.008	0.131	0	−0.014	0.196	0
Scio-economic status → Behaviors	− 0.009	0.160	0	−0.034	0.474	0.002

^**a**^***f*** ^**2**^ = 0.02, 0.15, and 0.35 as small, median, and large size of the effect, respectively

**Fig. 3:**
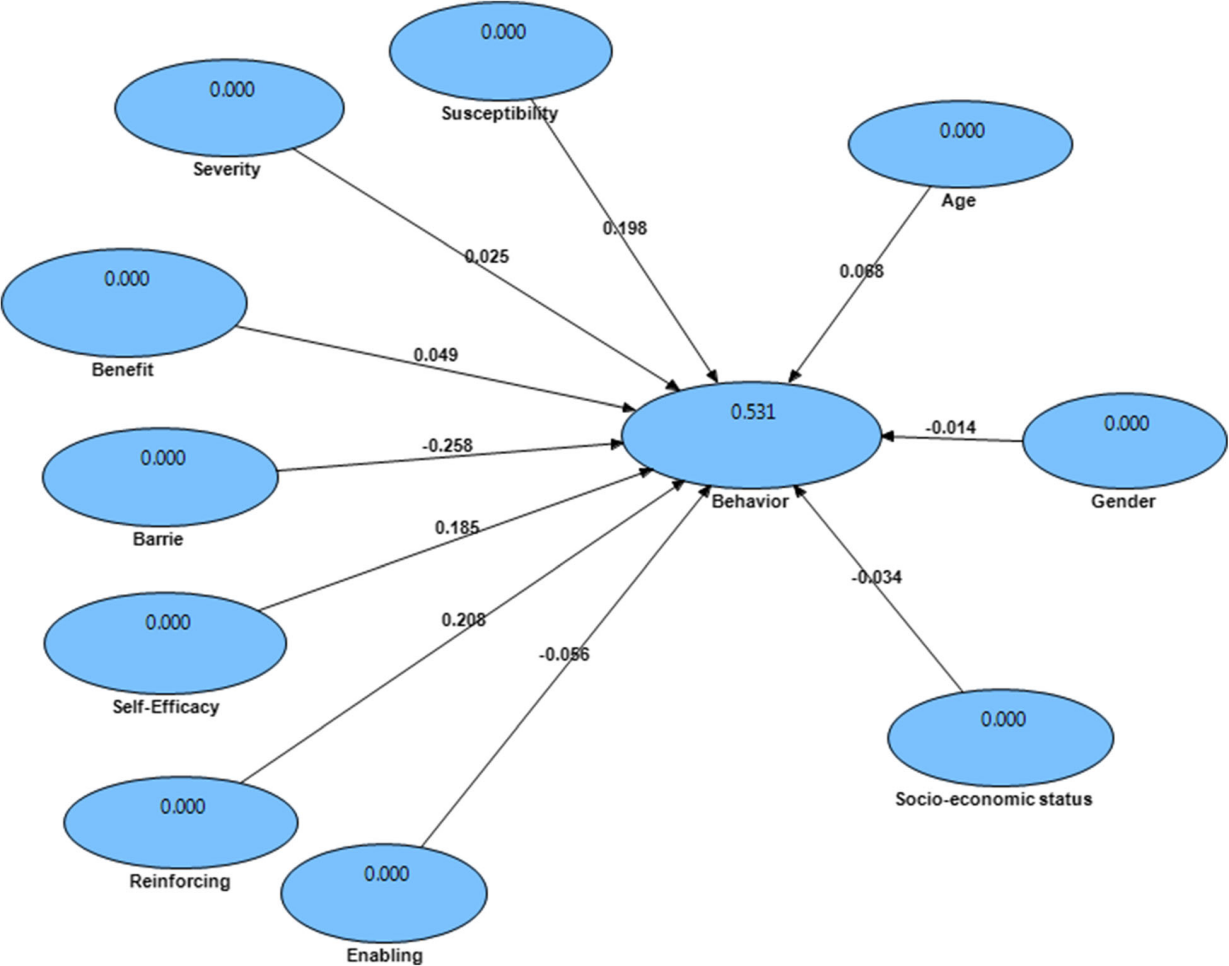
Structural model of backpack carrying behaviors in the prediction of path coefficients

Among predisposing factors for backpack carrying behaviors, the perceived susceptibility (*p* < 0.05, β = 0.198), perceived barriers (*p* < 0.05, β = − 0.258), perceived self-efficacy (*p* < 0.05, β = 0.185) and reinforcing factors (*p* < 0.05, β = 0.208) had a significant relationship with backpack carrying behaviors. However, perceived severity (*p* > 0.05, β = 0.025) and perceived benefits (> 0.05, β = 0.049) of predisposing factors as well as enabling factors (*p* > 0.05, β = − 0.056) age (*p* > 0.05, β = 0.068), gender (*p* > 0.05, β = − 0.014) and socio-economic status (*p* > 0.05, β = − 0.034) had no significant relationships with backpack carrying behaviors (Fig. 3). Also the size effect values are shown in Table 6.

The model’s predictive power was tested by calculating Q^2^ indexes to sitting behaviors (Q^2^ = 0.35) and backpack carrying behaviors (Q^2^ = 0.24), exceeding the recommended threshold value (Q^2^ > 0) [40], indicating an adequate predictive value of the model.

Finally, the goodness-of-fit (GOF) of the final model was evaluated. Wetzels et al. [41] suggested that GOF values above 0.36 indicate that the model is suitable for behavioral science. In the present study, the GOF model was estimated to be 0.63 for sitting behaviors and 0.55 for backpack carrying behavior.

## Discussion

The present study aimed to identify influential factors on behaviors related to musculoskeletal pains in students using models of PRECEDE and Health Belief. The levels of proper behaviors of backpack carrying and sitting postures were lower than average, so that 33 and 42% of the maximum possible score was for these behaviors respectively. Studies have shown that the poor postures and prolonged sitting are common in the classroom [42, 43]. Sezer et al. reported that 96.2% of the children wore their backpacks using both shoulder straps, 54.2% of their backpacks were not in full contact with their backs and 42.1% of the children wore their backpacks below their back and did not use a waist belt [44]. Barkhordari et al. reported that 83.4% of children carried their backpacks on both shoulders and only 4% used a wheeled [45]. Paula et al. found that the backpacks of 88.3% of children fully contacted their backs and only 6.33% used a waist belt [46].

Results indicated that reinforcing and predisposing factors (perceived susceptibility, perceived barriers, perceived self-efficacy) had a significant relationship with the behaviors related to carrying backpacks, and the sitting postures were significantly related to enabling and predisposing factors (perceived susceptibility, perceived severity, perceived barriers, perceived self-efficacy).

Model-based studies in different fields have shown that enabling factors are effective in forming appropriate behaviors [21, 47–49]. Students spend most of their hours in schools in sitting position. As an enabling factor, suitable furniture can reduce fatigue or uncomfortable sitting posture, which in turn leads to more concentration on learning [50]. However, a limited part of posture problems may be resolved by designing suitable furniture and training students on how to sit in a good position [7, 26, 51]. Therefore, ergonomic interventions (designing good furniture), along with education and exercise (stretching) seems necessary for posture improvement [52, 53].

On the other hand, in the present study, there was a significant relationship between backpack carrying behaviors and reinforcing factors. Other studies in the field of health have admitted the effectiveness of reinforcing factors in promoting healthy behaviors [48, 54]. The role of family, peers and teachers, as reinforcing factors, is outstanding in creating and continuing healthy behaviors [16, 55]. Therefore, in educational interventions, taking such influential groups and their roles is of great importance.

Results of the present study indicated that predisposing factors (perceived susceptibility, perceived severity, perceived barriers, and perceived self-efficacy) had significant relationships with good sitting postures. This significant relationship was also observed among backpack carrying behaviors with predisposing factors (perceived susceptibility, perceived barriers, and perceived self-efficacy). Self-efficacy influenced the start and the continuation of a behavior and modeling, feedback and reattribution are important factors of improved self-efficacy in behaviors related to health [56]. Teaching a behavior should be in a way that learners learn from models (alternative experiences) and direct successful experiences to believe that they have the ability to do the activities. The educations should also be in a way that students get the cognitive belief that they can be healthier by proper behaviors.

The significant relationship between constructs of perceived susceptibility and severity was another finding of this study. If people take negative consequences seriously, they will act to prevent those [22]. Features of society are therefore taken into account in designing educational plans.

Finally, perceived barriers had a significant relationship with good sitting postures and backpack carrying. Based on health belief model, barriers of promoting health behaviors like perceived unavailability, improperness, costs, nature of problems, and time-consuming of a certain behavior may be abstract or real [22]. Therefore, it is necessary for researchers and educators to consider abstract and real barriers and focus on the most important one to eliminate it in designing educational plans.

This study had several limitations that should be considered when interpreting the findings. First, the students were elementary, and they were likely to have problems in properly filling the questionnaire. However, interviews were used to enhance the accuracy of data. Second, one of the main limitations with self-report data is that students tend to give socially acceptable answers, that is, the elementary students may have tended to give responses making them look good. To reduce this effect, the students were explained about the importance of the study and giving accurate answers and also about the confidentiality of data and anonymity of the participants. As the third limitation, the study employed a cross-sectional data collection procedure and there is not possibility of finding causal inferences in the studies with this design. Also as the final limitation, it can be noted that in this study, the past medical and/or surgical history of participants were not captured.

## Conclusion

Various factors have effects on forming healthy behaviors in students. In the present study, the PRECEDE model and Health Belief model were used to identify behaviors of carrying backpack and sitting postures in elementary students; these models are good in the identification of factors influencing behaviors related to musculoskeletal pains in students. Regarding the identified factors for behaviors related to musculoskeletal pains in students predisposing, reinforcing and enabling factors have proved to be very important to be considered in future interventional plans, to have healthier populations.

## Data Availability

The datasets used and analyzed during the current study are available from the corresponding author on reasonable request.

## Abbreviations

MSK: Musculoskeletal
CVR: Content validity ratio
CVI: Content validity index
ICC: Interclass correlation coefficient
SEM: Structural equation modeling
AVE: Average variance extracted
GOF: Goodness-of-fit
