# Correction to: Identification of factors related to behaviors associated with musculoskeletal pain among elementary students

**DOI:** 10.1186/s12891-021-04526-9

**Published:** 2021-07-31

**Authors:** Forouzan Rezapur-Shahkolai, Elham Gheysvandi, Akram Karimi-Shahanjarini, Leili Tapak, Rashid Heidarimoghadam, Iman Dianat

**Affiliations:** 1 Department of Public Health, School of Public Health, Hamadan University of Medical Sciences, Hamadan, Iran; 2 Social Determinants of Health Research Center, Hamadan University of Medical Sciences, Hamadan, Iran; 3 Research Center for Health Sciences, Hamadan University of Medical Sciences, Hamadan, Iran; 4 Department of Biostatistics, School of Public Health, Hamadan University of Medical Sciences, Hamadan, Iran; 5 Modeling of Non-communicable Diseases Research Center, Hamadan University of Medical Sciences, Hamadan, Iran; 6 Department of Ergonomics, School of Public Health, Hamadan University of Medical Sciences, Hamadan, Iran; 7 Department of Occupational Health and Ergonomics, Faculty of Health, Tabriz University of Medical Sciences, Tabriz, Iran

**Correction to** **:** **BMC Musculoskelet Disord 22, 527 (2021)**

**https://doi.org/10.1186/s12891-021-04413-3**

Following the publication of the original article [1] the authors noticed that the components of Fig. 1 have been messed up. Below is the correct presentation of Fig. 1.

**Fig. 1:**
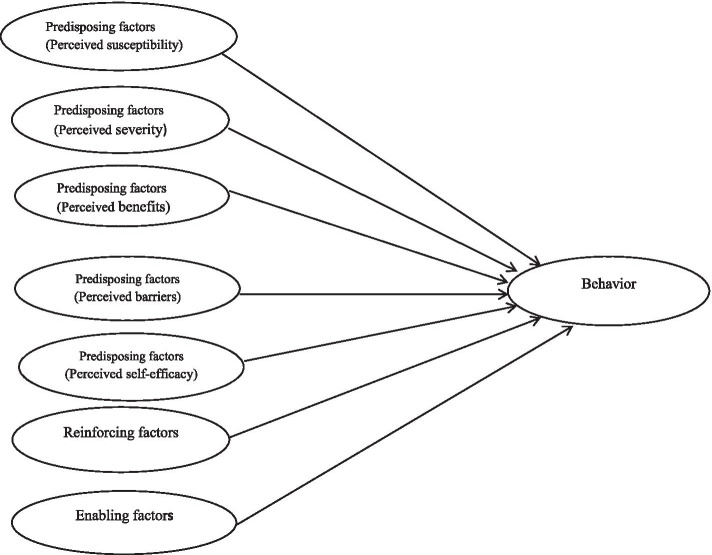
Conceptual framework of the model used in this study, employing PRECEDE and Health Belief Models

The original article [1] has been updated.
